# Bioactive Bibenzyl Enantiomers From the Tubers of *Bletilla striata*

**DOI:** 10.3389/fchem.2022.911201

**Published:** 2022-06-09

**Authors:** Mei Zhou, Sai Jiang, Changfen Chen, Jinyu Li, Huayong Lou, Mengyun Wang, Gezhou Liu, Hanfei Liu, Ting Liu, Weidong Pan

**Affiliations:** ^1^ School of Basic Medical Sciences/State Key Laboratory of Functions and Applications of Medicinal Plants, Guizhou Medical University, Guiyang, China; ^2^ The Key Laboratory of Chemistry for Natural Products of Guizhou Province and Chinese Academy of Sciences, Guiyang, China; ^3^ TCM and Ethnomedicine Innovation and Development International Laboratory, School of Pharmacy, Innovative Materia Medica Research Institute, Hunan University of Chinese Medicine, Changsha, China; ^4^ Guizhou Provincial Key Laboratory of Pharmaceutics, Guizhou Medical University, Guiyang, China

**Keywords:** *Bletilla striata*, bibenzyl enantiomers, antibacterial, anti-TNF-α activity, neuroprotection

## Abstract

Six new bibenzyls (three pairs of enantiomers), bletstrins D–F (1–3), were isolated from the ethyl acetate-soluble (EtOAc) extract of tubers of *Bletilla striata* (Thunb.) Rchb f. Their structures, including absolute configurations, were determined by 1D/2D NMR spectroscopy, optical rotation value, and experimental electronic circular dichroism (ECD) data analyses, respectively. Compounds 1–3 possess a hydroxyl-substituted chiral center on the aliphatic bibenzyl bridge, which represented the first examples of natural bibenzyl enantiomers from the genus of *Bletilla*. The antibacterial, antitumor necrosis factor (anti-TNF-*α*), and neuroprotective effects of the isolates have been evaluated. Compounds 3a and 3b were effective against three Gram-positive bacteria with minimum inhibitory concentrations (MICs) of 52–105 μg/ml. Compounds 2a and 2b exhibited significant inhibitory effects on TNF-*α*-mediated cytotoxicity in L929 cells with IC_50_ values of 25.7 ± 2.3 μM and 21.7 ± 1.7 μM, respectively. Subsequently, the possible anti-TNF-*α* mechanism of 2 was investigated by molecular docking simulation. Furthermore, the neuroprotective activities were tested on the H_2_O_2_-induced PC12 cell injury model, and compounds 2b, 3a, and 3b (10 μM) could obviously protect the cells with the cell viabilities of 57.86 ± 2.08%, 64.82 ± 2.84%, and 64.11 ± 2.52%, respectively.

## Introduction

The tubers of *Bletilla striata* (Thunb.) Rchb. f, named “Bai Ji”, is a traditional Chinese medicine, which are used for the treatment of several health disorders, including gastrointestinal disorders, ulcers, lung disorders, chapped skin, and traumatic bleeding (He et al., 2017; Zhang et al., 2019; Liao et al., 2019; Wang et al., 2020; Hou et al., 2021; Wang et al., 2021; Jiang et al., 2021; Xu et al., 2021). As an Orchidaceae plant, it can biosynthesize many secondary stilbenes, such as bibenzyls, phenanthrenes, dihydrophenanthrenes, biphenanthrenes, dihydrophenanthrofurans, and phenanthrenequinones (Sun et al., 2016; Jiang et al., 2019a; Zhu et al., 2021). Some of these compounds showed a wide range of biological activities like antibacterial, anti-inflammatory, neuroprotective, anticancer, and antiviral effects (Qian et al., 2015; Wang and Meng, 2015; Xu et al., 2019; Jiang et al., 2020; Jiang et al., 2021). In our previous work, more than 40 stilbenes including 13 new compounds were isolated from the tubers of *B. striata* and just tested for their antibacterial effects (Jiang et al., 2019a, Jiang et al., 2019b).

**FIGURE 1:**
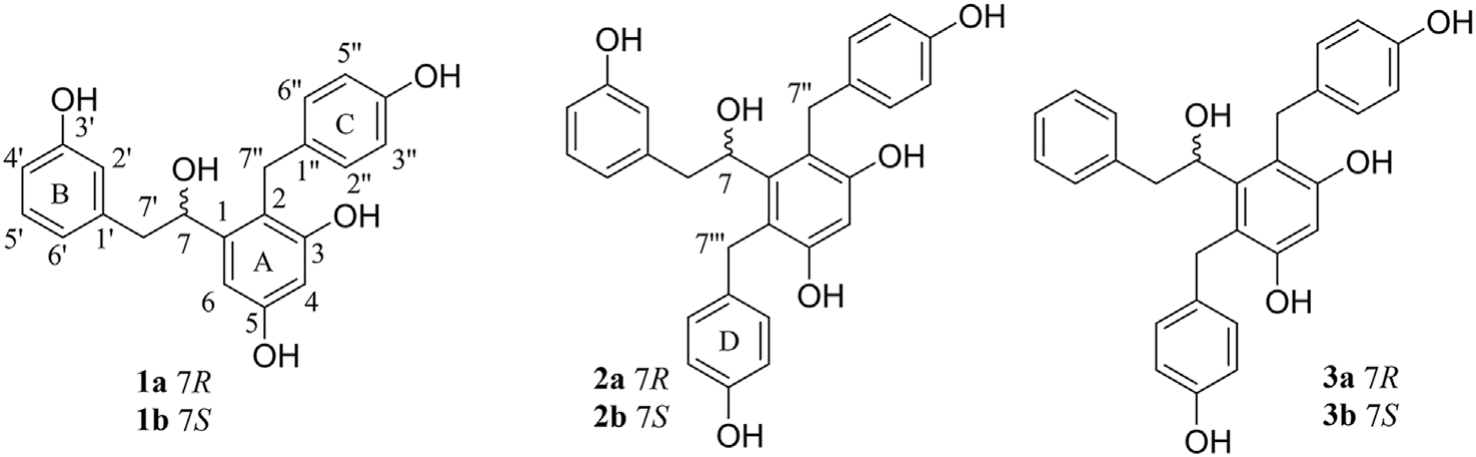
Isolated compounds 1–3 from the tubers of *B*. *striata.*

Some literature studies showed that some compounds isolated from the tubers of *B. striata* presented obvious anti-neuroinflammatory activities (Sun et al., 2021), indicating that *B. striata* might be a promising source of neuroprotection lead compounds. The aim of this study was to obtain further chemical and biological properties of *B. striata*, which might provide deeper insights into the plant as a promising Chinese medicine. As a result, six new bibenzyls (three pairs of enantiomers), bletstrins D-F (1–3), were isolated (Figure 1). The isolation and identification of these undescribed compounds and their absolute configurations were elucidated in this study. Moreover, all the compounds were tested for their antibacterial, anti-TNF-*α,* and neuroprotective activities. The molecular docking experiment was further conducted to reveal the potential mechanism of anti-TNF-*α* activity.

## Materials and Methods

### General Experimental Procedures

Optical rotations were measured on a Rudolph Autopol IV-T polarimeter equipped. The UV spectra were measured on an HP 8543E spectrometer. IR spectra were obtained on a Nicolet iS10 and an ICAN 9 FT-IR spectrometer with KBr pellets. ECD spectra were recorded with an Applied Photophysics Chirascan instrument. ^1^D and ^2^D NMR spectra were recorded on a Varian Inova 400 Hz NMR instrument or a Bruker Avance NEO 600 MHz spectrometer with tetramethylsilane (TMS) as the internal standard. The high-resolution electrospray ionization mass spectra (HRESIMS) were obtained on a Thermo Q-Exactive Focus mass spectrometer. All the solvents used were of analytical grade (Jiangsu Hanbang Science and Technology Co., Ltd.). Silica gel (300–400 mesh, Qingdao Haiyang Chemical Co., Ltd.), CHP20/P120 MCI gel (75–150 μm, Mitsubishi Chemical Industries, Ltd.), and Sephadex LH-20 (25–100 μm, Amersham Biosciences, Sweden) were used for column chromatography (CC). Semi-preparative HPLC was performed on a Waters-600 machine with a W2489 UV detector, column: ODS (5 μm, 10 × 250 mm, Waters Co., Ltd, United States). Chiral HPLC was performed on a Waters-600 machine with a W2489 UV detector equipped with a CHIRALPAK IA column (4.6 i. d. × 250 mm, S-5 μm, Daicel Chiral Technologies Co., Ltd., Japan). GF-254 (Qingdao Haiyang Chemical Co., Ltd.) was used for TLC.

### Plant Material

The tubers of *B. striata* were collected from Anlong County of Guizhou Province, People’s Republic of China, in March 2017, and authenticated by Prof. Ming-kai Wu (Institute of Modern Chinese Medicinal Materials, Guizhou Academy of Agricultural Sciences, Guizhou). A voucher specimen (No. 20170312003) was deposited at the State Key Laboratory of Functions and Applications of Medicinal Plants, Guizhou Medical University, People’s Republic of China.

### Extraction and Isolation

The dried tubers of *B. striata* (9.8 kg) were extracted with 95% ethyl alcohol (EtOH) under reflux four times to produce the crude extract. The residue was suspended in water and subsequently separated with EtOAc to yield an EtOAc-soluble fraction (386.1 g). The extract was purified on a silica gel column and eluted with a gradient CHCl_3_-CH_3_OH solvent system (100:1→0:1) to give 11 fractions (Frs. 1–11). Frs. 5 (25.2 g) was separated by MCI CC eluted with H_2_O-CH_3_OH (from 60:0 to 0:100) to yield nine subfractions (Frs. 5.1–5.9). Frs. 5.5 (1.3 g) was further separated on a silica gel column and eluted with petroleum ether (PE)-EtOAc (10:1 to 1:1) to give five subfractions (Frs. 5.5.1–5.5.5). Frs. 5.5.4 (113.2 mg) was purified by semi-preparative HPLC (CH_3_OH-H_2_O, 56:44, flow rate 2.0 ml/min) to afford three subfractions (16.8 mg, *t*_R_ = 19.61 min). Frs. 5.5.5 (238.6 mg) was purified by semi-preparative HPLC (CH_3_OH-H_2_O, 56:44, flow rate 2.0 ml/min) to afford three subfractions (5.4 mg, *t*_R_ = 31.63 min). Frs. 5.6 (756.3 mg) was subjected to being chromatographed on a silica gel column and eluted with PE-EtOAc (10:1 to 1:1) to give three subfractions (Frs. 5.6.1–5.6.3). Frs. 5.6.3 (86.4 mg) was purified by semi-preparative HPLC (CH_3_OH-H_2_O, 55:45, flow rate 2.0 ml/min) to afford 1 subfraction (7.6 mg, *t*_R_ = 24.52 min). Compounds 1–3 were further purified by chiral HPLC, using CH_3_OH-H_2_O (50:50) as the mobile phase, to yield 1a (2.2 mg), 1b (2.8 mg), 2a (1.60 mg), 2b (1.85 mg), 3a (6.38 mg), and 3b (5.58 mg).

Bletstrin D (1) racemic mixture. Yellowish amorphous powder; UV (MeOH) *λ*_max_ (log *ε*) 280 (3.49) nm; IR (KBr): *ν*_max_ = 3,374, 1,611, 1,513, 1,225, 1,145, 986, and 693 cm^−1^; ^1^H and ^13^C NMR data (see Table 1; HRESIMS *m/z* 351.1241 [M − H]^−^ (calcd. for C_21_H_19_O_5_, 351.1232).

**TABLE 1:** ^1^H and^13^C NMR data of compounds 1–3.

	1^a^		2^b^		3^c^	
No.	*δ*_C_	*δ*_H_ (*J* in Hz)	*δ*_C_	*δ*_H_ (*J* in Hz)	*δ*_C_	*δ*_H_ (*J* in Hz)
1	146.7		144.0		144.0	
2	114.4		117.9		117.7	
3	155.7		155.3		155.4	
4	100.9	6.23, d (2.4)	103.0	6.43, s	102.7	6.45, s
5	156.1		156.9		156.9	
6	104.0	6.49, d (2.4)	118.8		118.8	
7	70.0	4.76, dd (10.8 and 6.0)	73.6	5.17, dd (8.8 and 4.8)	73.7	5.15, dd (9.2 and 4.4)
1′	141.2		142.4		140.9	
2′	116.3	6.58, m	117.4	6.41, m	130.5	6.72, m
3′	156.9		157.9		128.9	7.11, m
4′	112.7	6.55, m	113.7	6.53, m	126.8	7.08, m
5′	128.7	6.99, t (7.8)	129.9	6.94, m	128.9	7.11, m
6′	120.0	6.46, m	121.8	6.21, m	130.5	6.76, m
7′	45.3	2.47, d (6.0)	44.3	2.70, dd (13.6 and 8.8)	44.3	2.77, dd (13.6 and 9.2)
				2.34, dd (13.6 and 4.8)		2.35, dd (13.6 and 4.4)
1″	131.8		134.3		134.3	
2″, 6″	128.9	6.89, d (8.4)	130.1	6.94, m	130.1	6.94, m
3″, 5″	114.8	6.60, d (8.4)	115.6	6.66, d (8.4)	115.6	6.67, m
4″	155.0		155.5		155.5	
7″	28.8	3.76, d (15.6)	30.8	3.83, d (16.0)	30.8	3.78, s
		3.68, d (15.6)		3.69, d (16.0)		
1‴			135.2		135.2	
2‴, 6‴			130.1	6.94, m	130.1	6.94, m
3‴, 5‴			115.9	6.66, d (8.4)	115.9	6.67, m
4‴			156.0		156.1	
7‴			31.6	4.56, d (16.0)	31.7	4.57, d (15.6)
				4.18, d (16.0)		4.20, d (15.6)
1′′′′						
2′′′′, 6′′′′						
3′′′′, 5′′′′						
4′′′′						
7′′′′						
3-OH		9.07, s				
5-OH		8.96, s				
7-OH		4.85, d (4.2)				
3′-OH		9.03, s or 9.16, s				
4″-OH		9.16, s or 9.03, s				

a ^1^H (600 MHz) and ^13^C (150 MHz) NMR, data on DMSO-*d*_6._

b ^1^H (400 MHz) and ^13^C (100 MHz) NMR, data on methanol-*d*_4._

c ^1^H (400 MHz) and ^13^C (100 MHz) NMR, data on methanol-*d*_4._

(7*R*)-bletstrin D (1a). ${\left[\alpha \right]}_{D}^{22}$ +18.3 (*c* 0.04, MeOH); ECD (MeOH) *λ*_max_ (Δ*ε*) 206 (+2.97), 219 (−0.74), and 236 (+1.03) nm.

(7*S*)-bletstrin D (1b). ${\left[\alpha \right]}_{D}^{22}$ −2.1 (*c* 0.05, MeOH); ECD (MeOH) *λ*_max_ (Δ*ε*) 206 (−2.24), 217 (+0.50), and 236 (−0.68) nm.

Bletstrin E (2) racemic mixture. Yellowish amorphous powder; UV (MeOH) *λ*_max_ (log *ε*) 280 (2.93) nm; IR (KBr): *ν*_max_ = 3,381, 1,595, 1,510, 1,457, 1,236, and 1,172 cm^−1^; ^1^H and ^13^C NMR data (see Table 1); HRESIMS *m/z* 457.1664 [M − H]^−^ (calcd. for C_28_H_25_O_6_, 457.1651).

(7*R*)-bletstrin E (2a). ${\left[\alpha \right]}_{D}^{22}$ +12.5 (*c* 0.05, MeOH); ECD (MeOH) *λ*_max_ (Δ*ε*) 202 (+6.49), 217 (−1.51), and 230 (+1.13) nm.

(7*S*)-bletstrin E (2b). ${\left[\alpha \right]}_{D}^{22}$ −4.5 (*c* 0.06, MeOH); ECD (MeOH) *λ*_max_ (Δ*ε*) 201 (−3.97), 218 (+1.28), and 230 (−0.51) nm.

Bletstrin F (3) racemic mixture. Yellowish amorphous powder; UV (MeOH) *λ*_max_ (log *ε*) 285 (3.23) nm; IR (KBr): *ν*_max_ = 3,379, 1,599, 1,510, 1,238, 1,170, 1,083, and 702 cm^−1^; ^1^H and ^13^C NMR data (see Table 1); HRESIMS *m/z* 443.1852 [M + H]^+^ (calcd. for C_28_H_27_O_5_, 443.1858).

(7*R*)-bletstrin F (3a). ${\left[\alpha \right]}_{D}^{22}$ +28.7 (*c* 0.05, MeOH); ECD (MeOH) *λ*_max_ (Δ*ε*) 203 (+4.11), 219 (−1.07), and 231 (+1.35) nm.

(7*S*)-bletstrin F (3b). ${\left[\alpha \right]}_{D}^{22}$ −10.1 (*c* 0.05, MeOH); ECD (MeOH) *λ*_max_ (Δ*ε*) 204 (−2.24), 219 (+1.38), and 231 (−0.54) nm.

### Antibacterial Activity Assays

Antimicrobial activities of compounds 1–3 against Gram-positive bacteria (Methicillin-resistant *S. aureus* ATCC 43300, *S. aureus* ATCC 6538, and *Bacillus subtilis* ATCC 6051) and Gram-negative bacteria (*Escherichia coli* ATCC 11775) were performed using a microbroth dilution method in a 96-well microtiter plate (Jiang, et al., 2019a). Bacteria were seeded at 1 × 10^6^ cells per well (200 μL) in a 96-well plate containing Mueller-Hinton broth with different concentrations (from 1 to 420 μg/ml; 2-fold increments) of each test compound. Oxacillin, which was obtained from J&K Chemicals (Beijing, China), was used as a positive control.

### Anti-TNF-α Activity Assay

L929 cells (Procell, Wuhan, China) were cultured in RPMI 1640 (Gibco, United States) supplemented with 10% fetal calf serum (Procell, Wuhan, China) at 37°C in a humidified atmosphere of 5% CO_2_. Exponentially growing L929 cells were harvested and seeded in 96-well multiplates at a density of 1.5 × 10^5^ cells/mL. After incubation for 24 h at 37°C, samples (0.01, 0.1, 10, 20, 40, 80, and 200 μM), TNF-α (GlpBio, Shanghai, China) (7.5 ng/ml), and actinomycin D (GlpBio, Shanghai, China) (0.5 μg/ml) were added. After 12 h incubation at 37°C, 100 μL of 3-(4,5-dimethylthiazol-2-yl)-5-2 -(4-sulfophenyl)-2H-tetrazolium (MTS) (Promega, United States) (0.5 mg/ml) was added to each well and incubated for an additional 2 h. The optical density (OD) of the formazan solution was measured using a microplate reader at 490 nm. UCB-9260 (GlpBio, Shanghai, China) was used as a positive control.

### Neuroprotective Activity Assay

PC12 cells were cultured in Ham’s F12K (Gibco, United States) with 10% fetal calf serum at 37°C in a humidified atmosphere of 5% CO_2_. The cells were seeded in 96-well multiplates at a density of 1.5 × 10^5^ cells/mL. After overnight incubation at 37°C with 5% CO_2_, 10 μM test samples and H_2_O_2_ (final concentration of 450 μM) were added into the wells and incubated for another 12 h. The cell survival rate was measured by 3-(4,5-dimethylthiazol-2-yl)-2,5-diphenyltetrazolium bromide (MTT) (Gibco, United States) assay (Chu et al., 2019).

## Results and Discussion

**FIGURE 2:**
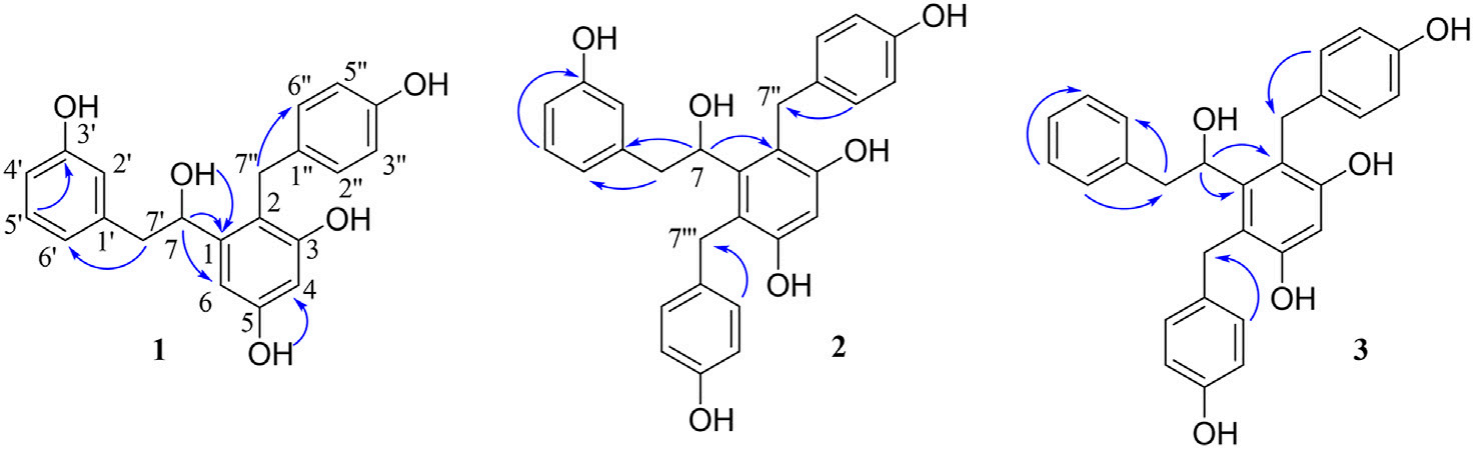
Key HMBC correlations of compounds 1–3.

Compound 1 was obtained as a yellowish amorphous powder. HRESIMS analysis established the molecular formula of 1 as C_21_H_20_O_5_ (*m/z* 351.1241 [M - H]^−^, calcd. 351.1232). The UV spectrum suggested the presence of aromatic ring functional groups. The ^1^H NMR spectrum of 1 revealed signals for one 1,3-disubstituted phenyl group [*δ*_H_ 6,99 (1H, t, *J* = 7.8 Hz), 6.58 (1H, m), 6.55 (1H, m), and 6.46 (1H, m)]; one 4-hydroxybenzyl moiety [*δ*_H_ 6.89 (2H, d, *J* = 8.4 Hz), 6.60 (2H, d, *J* = 8.4 Hz), 3.76 (1H, d, *J* = 15.6 Hz), and 3.68 (1H, d, *J* = 15.6 Hz)]; one 1,2,3,5-tetrasubstituted aromatic moiety [*δ*_H_ 6.49 (1H, d, *J* = 2.4 Hz) and 6.23 (1H, d, *J* = 2.4 Hz)]; one methylene group [*δ*_H_ 2.47 (2H, d, *J* = 6.0 Hz)]; one oxymethine group [*δ*_H_ 4.22 (1H, dd, *J* = 10.8, 6.0 Hz)]; and four hydroxy groups [*δ*_H_ 9.16 (1H, s), 9.07 (1H, s), 8.96 (1H, s), and 4.85 (1H, d, *J* = 4.2 Hz)]. The ^13^C NMR spectrum of 1 showed 21 signals, four of them were oxygen-bearing aromatic carbons (*δ*_C_ 156.9, 156.1, 155.7, and 155.0), and one of them was oxygen-bearing methylene group (*δ*_C_ 70.0). The 1D-NMR data of **1** were similar to those of bletstrin A (Jiang et al., 2019b), except for the existence of an extra hydroxy group linked to the C-3′ position. This conclusion was supported by the molecular weight and the HMBC (Figure 2) correlations of H-5′ (*δ*_H_ 6.99) and H-2′ (*δ*_H_ 6.58) to C-3′ (*δ*_C_ 156.9). Thus, the planar structure of **1** was established.

However, compound 1 was not optically pure but racemic according to its optical rotation data (Shao et al., 2019). We then separated optically pure compounds 1a and 1b from one by chiral HPLC. Compounds 1a and 1b exhibited opposite cotton effects at 206, 219, and 236 nm and further confirmed their racemic relationship. By comparison of the experimental ECD curves and data from the literature (Jiang et al., 2019b), the absolute configurations of 1a and 1b were determined as 7*R* and 7*S*, respectively. Finally, the structures of bletstrin D (1a and 1b) were defined. All the spectroscopic data of compound 1 are shown in Supplementary Figures S1–8 in Supplementary Material S1.

Compound 2 was obtained as a yellowish amorphous powder. Its molecular formula of C_28_H_26_O_6_ was determined by the (-)-HRESIMS ion peak at *m/z* 457.1644 [M - H]^−^ (calcd. 457.1651). The comparison of the ^1^D NMR data (Table 1) of 2 with those of 1 suggested that their structures were similar, except for the existence of an extra benzene moiety in 2. The ^1^H NMR data of compound 2 showed an extra AA′BB’ system benzene moiety at *δ*_H_ 6.94 (2H, m, H-2‴, 6‴), 6.66 (2H, d, *J* = 8.4 Hz, H-3‴, 5‴), 4.56 (1H, d, *J* = 16.0 Hz), and 4.18 (1H, d, *J* = 16.0 Hz) in the A ring. The ^13^C NMR spectrum of compound 2 showed seven extra signals at *δ*_C_ 135.2 (C-1‴), 130.1 (C-2‴), 115.6 (C-3‴), 155.5 (C-4‴), 115.6 (C-5‴), 130.1 (C-6‴), and 31.6 (C-7‴). Furthermore, the HMBC (Figure 2) correlations from H-7‴ (*δ*_H_ 4.56) to C-2‴, C‴, C-1, C-5, and C-6 suggested that the benzyl group was connected to C-6. Thus, the planar structure of 2 was defined and named as bletstrin E. Compound 2 was also a racemic mixture. Resolution by chromatography analysis is afforded to 2a and 2b. The absolute configurations of 2a and 2b were determined as 7*R* and 7*S* by comparing with the experimental ECD spectra of 1a and 1b. Finally, the structures of bletstrin E (2a and 2b) were defined. All the spectroscopic data on compound 2 are shown in Supplementary Figures S8–16 in Supplementary Material S1.

**FIGURE 3:**
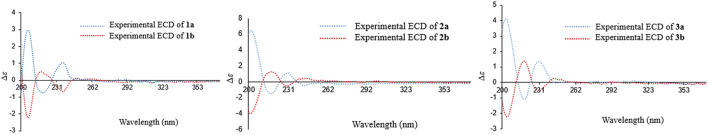
Experimental ECD curves of compounds 1–3.

Compound 3, a yellowish amorphous powder, was given the molecular formula of C_28_H_26_O_5_ by (+)-HRESIMS ion peak at *m/z* 443.1852 [M + H]^+^ (calcd. 443.1858). A comparison of the molecular formula of 3 with that of 2 inferred that it lacks an oxygen atom. By detailed analysis of the 1D NMR data (Table 1) of 3, it was suggested that its structure was similar to that of **2**, except for missing a hydroxy group at position C-3′. The location was supported by the downfield chemical shift of C-3′ at *δ*_C_ 128.9 (Δ*δ*_C_ −29.0), as well as the HMBC (Figure 2) correlations of H-3′, 5′ (*δ*_H_ 7.11) with C-1′ (*δ*_C_ 140.9). Compound 3 was a racemate according to its optical rotation data. The pair of optically pure enantiomers (3a and 3b) was separated using chiral chromatography analysis. The absolute configurations of 3a and 3b were determined as 7*R* and 7*S* by comparing with the experimental ECD (Figure 3) spectra of 1a and 1b. Finally, the structures of bletstrin F (3a and 3b) were defined. All the spectroscopic data on compound 3 are shown in Supplementary Figures S17–24 in Supplementary Material S1.

**FIGURE 4:**
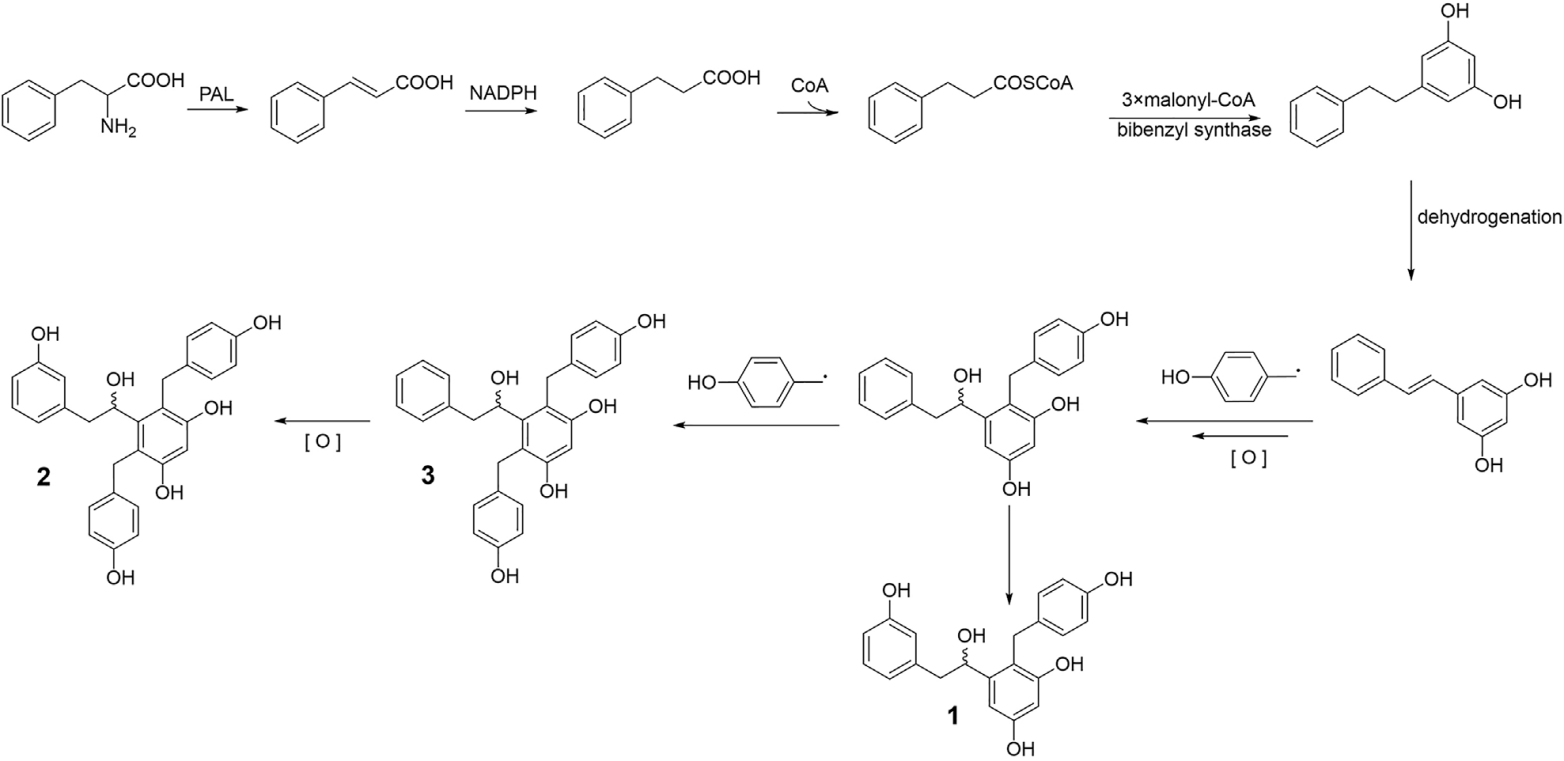
Plausible biogenetic pathway of compounds 1–3.

Bletstrins D–F (1–3) are all new bibenzyl derivatives possessing a rare hydroxyl substituted chiral center on the aliphatic bibenzyl bridge structure. In the field of a biosynthetic pathway, the establishment of the new structures may consist of a series of modifications from simple model blocks (Sun et al., 2021). As shown in Figure 4, bibenzyls are biosynthesized from dihydro-m-coumaroyl-CoA (Jiang et al., 2019). Subsequently, the target compounds 1, 2, and 3 were formed by dehydrogenation, oxidation, and the coupling of one or two benzyl groups.

**FIGURE 5:**
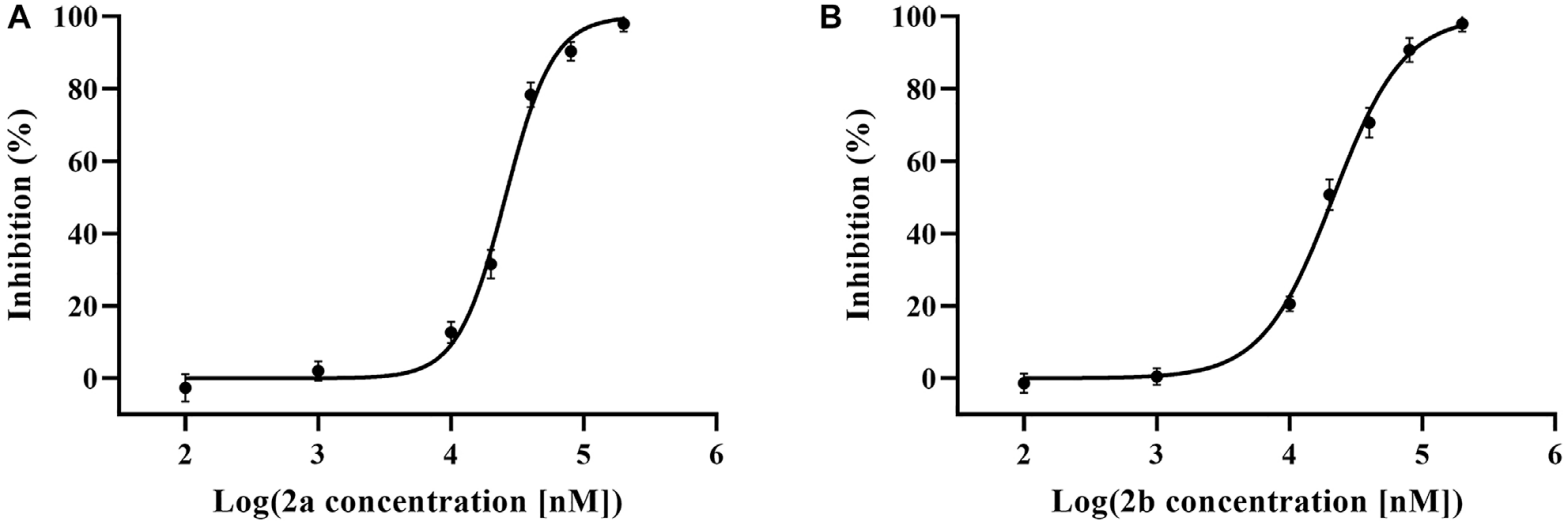
L929 assay of compound 2 **(A,B)** in the presence of different concentrations (*n* = 3).

**FIGURE 6:**
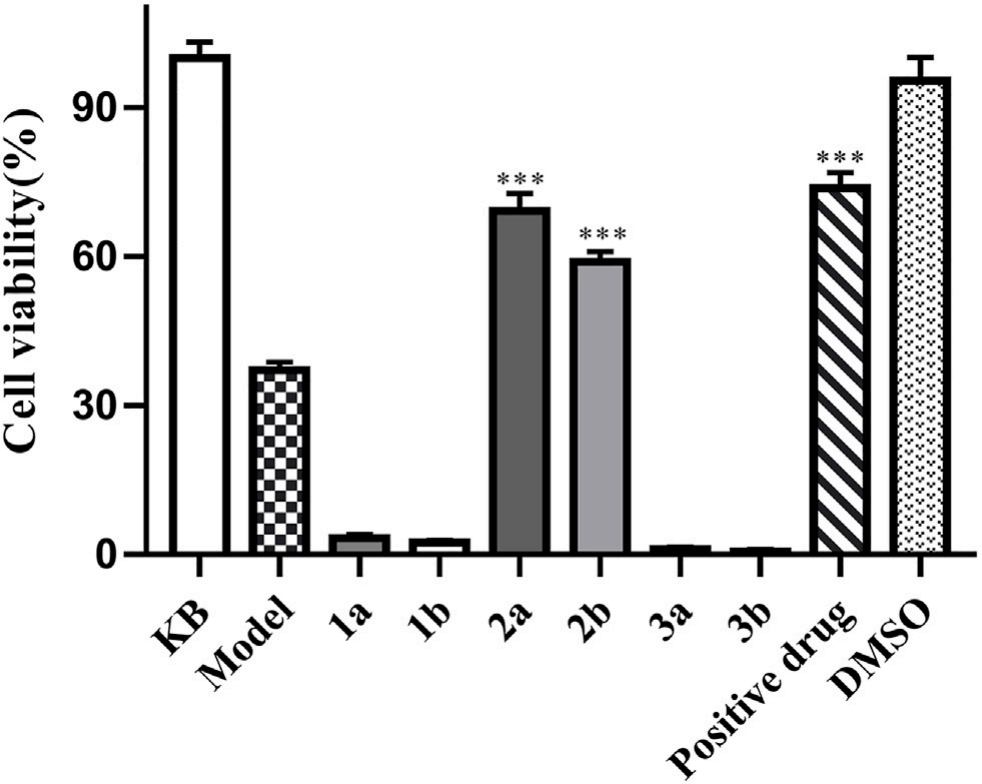
Anti-TNF-α activities of compounds 1–3. L929 cells were cultured in the presence of isolated compounds and were measured by the MTS assay (*n* = 3). Data are expressed as mean ± S D. ***p < 0.001 vs. the model group.

**FIGURE 7:**
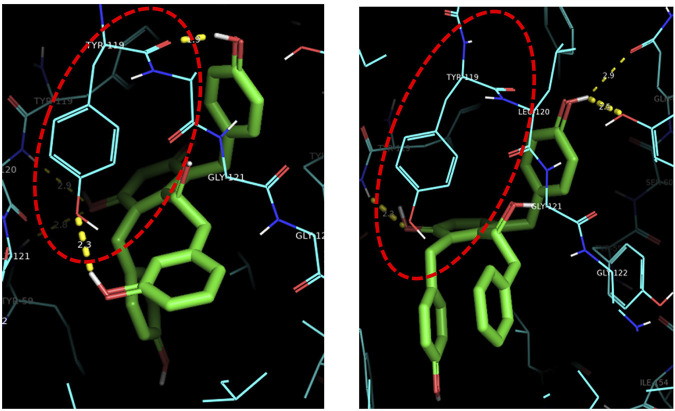
Three-dimensional molecular docking model of compounds 2 and 3. Detail of the compound-binding pocket within the TNF homotrimer, with key residues involved in binding highlighted.

Compounds 1–3 were tested on antibacterial activities against three common Gram-positive bacterial strains (Methicillin-resistant *Staphylococcus aureus* ATCC 43300, *S. aureus* ATCC 6538, and *Bacillus subtilis* ATCC 6051) and one Gram-negative bacterial strain (*Escherichia coli* ATCC 11775). The results revealed that compounds 3a and 3b were effective against three Gram-positive bacteria with MICs of 52–105 μg/ml (Supplementary Table S1 in Supplementary Material S1). Furthermore, compounds 2a and 2b exhibited obvious anti-TNF-*α* bioactivity in TNF-*α*-mediated-cytotoxicity assay with IC_50_ values of 25.7 ± 2.3 μM and 21.7 ± 1.7 μM, respectively (Figure 5); these values are of the same order of magnitude to the published literature (He et al., 2005; Alexiou et al., 2014; Melagraki et al., 2017). The cell viability of 69.17 ± 2.42% and 58.89 ± 2.08% at 20 μM, compared to the model group (37.08 ± 1.68%), while that of the positive control UCB-9260 was 73.70 ± 3.12% (Figure 6). TNF-α is a proinflammatory cytokine that plays a key role in most of the inflammatory processes (Jacobi et al., 2006), and these results suggested that compounds 2a and 2b may have obvious anti-inflammatory activity. In the anti-TNF-*α* activity test, compound 2, which possesses one more hydroxy group than compound 3 at C-4′, showed significantly different activities (*P* ˂ 0.001). The possible anti-TNF-*α* mechanisms of 2 and 3 were investigated by molecular docking simulation (Figure 7). From the docking mode of two compounds with TNF-α protein (PDB ID: 6OOY), it can be seen that compound 2 with hydroxy group at C-4′ can form two stable hydrogen bonds with 6OOY tyrosine at position 119 (tyrosine; Tyr), which could not be found in the docking model of 3 and 6OOY. In the published study on the crystal structure of 6OOY, it was also demonstrated that the ligand binds to the protein at position 119 of the B chain (O'Connell et al., 2019). This docking simulation revealed that compound 2 might form hydrogen bonds with amino acids at position 119 of the B chain of TNF-α, which can inhibit its activity by stabilizing the asymmetric trimer structure of TNF-α.

**FIGURE 8:**
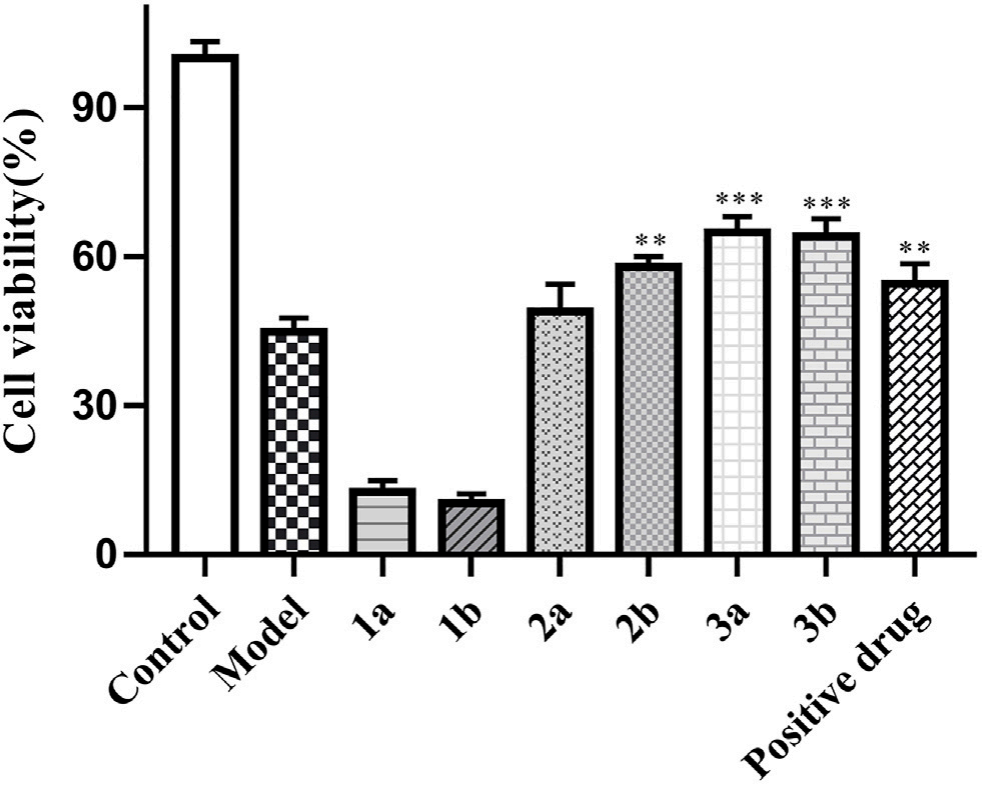
Neuroprotective activities of compounds 1–3. PC12 cells were in the presence of isolated compounds at a concentration of 10 μM with the H_2_0_2_-induced injury model. Their cell viabilities were measured by the MTT assay (*n* = 3). Data are expressed as mean ± S D. ***p < 0.001 vs. the model group.

Moreover, *in vitro* assays, compounds 2b, 3a, and 3b (10 μM) exhibited excellent neuroprotective activities against H_2_O_2_-induced PC12 cell damage with the cell viabilities of 57.86 ± 2.08%, 64.82 ± 2.84%, and 64.11 ± 2.52%, respectively, while that of the positive control (±) *α*-Tocopherol was 54.51 ± 2.87% (Figure 8).

## Conclusion

Six new bibenzyls (three pairs of enantiomers, 1a–3b) were isolated from the tubers of *B. striata*. The absolute configurations of compounds 1–3 were assigned by comparison of the optical rotation value combined with their experimental ECD data. All the compounds were evaluated for their antibacterial activities, but only compound 3 showed inhibitory activities against the three Gram-positive bacteria. This result preliminarily inferred that the orientation of hydroxyl at C-7 may not affect the antibacterial activities. Furthermore, the anti-TNF-*α* activity was influenced by the hydroxyl at C-4′ and the benzyl at C-6 by comparing the effects of 1, 2, and 3. Moreover, the absolute configurations may also not affect their neuroprotective activities, but the benzyl group at C-6 may affect their activity. In fact, this is the first example of natural bibenzyl enantiomers (a hydroxyl-substituted chiral center on the aliphatic bibenzyl bridge) from the genus of *Bletilla*. Furthermore, the anti-TNF-α and neuroprotective effects of bibenzyls from *B. striata* are also the first reported. In addition, some simple bibenzyls (not containing extra benzyl groups) from *Stemona* and *Dendrobium* species also showed neuroprotective activities against 6-hydroxydopamine-induced neurotoxicity in human neuroblastoma SH-SY5Y cells (Lee et al., 2006; Song et al., 2010). In summary, according to the structure and activity relationship (SAR), the hydroxyl on the aliphatic chain and/or benzyl groups, as well as the absolute configurations, may affect the bioactivities of bibenzyls.

## Data Availability

The original contributions presented in the study are included in the article/Supplementary Material; further inquiries can be directed to the corresponding authors.

## References

[B1] Alexiou P. Papakyriakou A. Ntougkos E. Papaneophytou C. P. Liepouri F. Mettou A. (2014). Rationally Designed Less Toxic SPD-304 Analogs and Preliminary Evaluation of Their TNF Inhibitory Effects. Arch. Pharm. Chem. Life Sci. 347, 798–805. 10.1002/ardp.201400198 25160057

[B2] Chu Q. Chen M. Song D. Li X. Yang Y. Zheng Z. (2019). Apios Americana Medik Flowers Polysaccharide (AFP-2) Attenuates H2O2 Induced Neurotoxicity in PC12 Cells. Int. J. Biol. Macromol. 123, 1115–1124. 10.1016/j.ijbiomac.2018.11.078 30445092

[B3] He M. M. Smith A. S. Oslob J. D. Flanagan W. M. Braisted A. C. Whitty A. (2005). Small-Molecule Inhibition of TNF-α. Science 310, 1022–1025. 10.101126/science.111630410.1126/science.1116304 16284179

[B4] He X. Wang X. Fang J. Zhao Z. Huang L. Guo H. (2017). Bletilla Striata: Medicinal Uses, Phytochemistry and Pharmacological Activities. J. Ethnopharmacol. 195, 20–38. 10.1016/j.jep.2016.11.026 27865796

[B5] Hou X.-Y. Cao Y. Wu B.-L. Chen B. Li F. Wang F. (2021). New 2-isobutylmalates from the Tubers of *Bletilla Striata* and Their Potential Anti-pulmonary Fibrosis Activities. Phytochem. Lett. 46, 95–99. 10.1016/j.phytol.2021.09.009

[B6] Jacobi A. Mahler V. Schuler G. Hertl M. (2006). Treatment of Inflammatory Dermatoses by Tumour Necrosis Factor Antagonists. J. Eur. Acad. Dermatol Venerol. 20 (10), 1171–1187. 10.1111/j.1468-3083.2006.01733.x 17062028

[B7] Jiang S. Chen C.-F. Ma X.-P. Wang M.-Y. Wang W. Xia Y. (2019a). Antibacterial Stilbenes from the Tubers of *Bletilla Striata* . Fitoterapia 138, 104350. 10.1016/j.fitote.2019.104350 31473333

[B8] Jiang S. Wan K. Lou H.-Y. Yi P. Zhang N. Zhou M. (2019b). Antibacterial Bibenzyl Derivatives from the Tubers of Bletilla Striata. Phytochemistry 162, 216–223. 10.1016/j.phytochem.2019.03.2210.1016/j.phytochem.2019.03.022 30953908

[B9] Jiang S. Wang M. Jiang L. Xie Q. Yuan H. Yang Y. (2021). The Medicinal Uses of the Genus *Bletilla* in Traditional Chinese Medicine: A Phytochemical and Pharmacological Review. J. Ethnopharmacol. 280, 114263. 10.1016/j.jep.2021.114263 34144194

[B10] Jiang S. Wang M. Y. Yuan H. W. Xie Q. Liu Y. Jian Y. Q. (2020). Medicinal Plant of *Bletilla Striata:* A Review of its Chemical Constituents, Pharmacological Activities, and Quality Control. World J. Tradit. Chin. Med. 6, 393–407. 10.4103/wjtcm.wjtcm5820

[B11] Lee K. Y. Sung S. H. Kim Y. C. (2006). Neuroprotective Bibenzyl Glycosides of *Stemona Tuberosa* Roots. J. Nat. Prod. 69, 679–681. 10.1021/np0504154 16643052

[B12] Liao Z. Zeng R. Hu L. Maffucci K. G. Qu Y. (2019). Polysaccharides from Tubers of *Bletilla Striata*: Physicochemical Characterization, Formulation of Buccoadhesive Wafers and Preliminary Study on Treating Oral Ulcer. Int. J. Biol. Macromol. 122, 1035–1045. 10.1016/j.ijbiomac.2018.09.050 30227203

[B13] Melagraki G. Ntougkos E. Rinotas V. Papaneophytou C. Leonis G. Mavromoustakos T. (2017). Cheminformatics-aided Discovery of Small-Molecule Protein-Protein Interaction (PPI) Dual Inhibitors of Tumor Necrosis Factor (TNF) and Receptor Activator of NF-κB Ligand (RANKL). PLoS Comput. Biol. 13, e1005372. 10.1371/journal.pcbi.1005372 28426652 PMC5398486

[B14] O'Connell J. Porter J. Kroeplien B. Norman T. Rapecki S. Davis R. (2019). Small Molecules that Inhibit TNF Signalling by Stabilising an Asymmetric Form of the Trimer. Nat. Commun. 10, 5795–5805. 10.1038/s41467-019-1316-110.1038/s41467-019-13616-1 31857588 PMC6923382

[B15] Qian C.-D. Jiang F.-S. Yu H.-S. Shen Y. Fu Y.-H. Cheng D.-Q. (2015). Antibacterial Biphenanthrenes from the Fibrous Roots of *Bletilla Striata* . J. Nat. Prod. 78, 939–943. 10.1021/np501012n 25760525

[B16] Shao S.-Y. Wang C. Han S.-W. Sun M.-H. Li S. (2019). Phenanthrenequinone Enantiomers with Cytotoxic Activities from the Tubers of *Pleione Bulbocodioides* . Org. Biomol. Chem. 17, 567–572. 10.1039/C8OB02850H 30574990

[B17] Song J.-X. Shaw P.-C. Sze C.-W. Tong Y. Yao X.-S. Ng T.-B. (2010). Chrysotoxine, a Novel Bibenzyl Compound, Inhibits 6-hydroxydopamine Induced Apoptosis in SH-SY5Y Cells via Mitochondria Protection and NF-κB Modulation. Neurochem. Int. 57, 676–689. 10.1016/j.neuint.2010.08.007 20708055

[B18] Sun A. Liu J. Pang S. Lin J. Xu R. (2016). Two Novel Phenanthraquinones with Anti-cancer Activity Isolated from Bletilla Striata. Bioorg. Med. Chem. Lett. 26, 2375–2379. 10.1016/j.bmcl.2016.01.076 26995526

[B19] Sun M.-H. Ma X.-J. Shao S.-Y. Han S.-W. Jiang J.-W. Zhang J.-J. (2021). Phenanthrene, 9,10-dihydrophenanthrene and Bibenzyl Enantiomers from *Bletilla Striata* with Their Antineuroinflammatory and Cytotoxic Activities. Phytochemistry 182, 112609. 10.1016/j.phytochem.202011260910.1016/j.phytochem.2020.112609 33326906

[B20] Wang B. Zhang H. Chen L. Mi Z. Xu Y. Zhao G. (2020). Extraction, Purification, and Determination of the Gastroprotective Activity of Glucomannan from *Bletilla Striata* . Carbohydr. Polym. 246, 116620. 10.1016/j.carbpol.202011662010.1016/j.carbpol.2020.116620 32747259

[B21] Wang W. Meng H. (2015). Cytotoxic, Anti-inflammatory and Hemostatic Spirostane-Steroidal Saponins from the Ethanol Extract of the Roots of *Bletilla Striata* . Fitoterapia 101, 12–18. 10.16/j.fitote.2014.11.00510.1016/j.fitote.2014.11.005 25447157

[B22] Wang X. Xing M. Zhang Z. Deng L. Han Y. Wang C. (2021). Using UPLC-QTOF/MS and Multivariate Analysis to Explore the Mechanism of *Bletilla Striata* Improving PM2.5-induced Lung Impairment. Anal. Biochem. 631, 114310. 10.1016/j.ab.2021.114310 34280371

[B23] Xu D. Pan Y. Chen J. (2019). Chemical Constituents, Pharmacologic Properties, and Clinical Applications of Bletilla Striata. Front. Pharmacol. 10, 1168–1186. 10.3389/fphar.2019.01168 31736742 PMC6838137

[B24] Xu J. Chen Z. Liu P. Wei Y. Zhang M. Huang X. (2021). Structural Characterization of a Pure Polysaccharide from Bletilla Striata Tubers and its Protective Effect against H2O2-Induced Injury Fibroblast Cells. Int. J. Biol. Macromol. 193, 2281–2289. 10.16/j.biomac.2021.11.06010.1016/j.ijbiomac.2021.11.060 34785199

[B25] Zhang C. Gao F. Gan S. He Y. Chen Z. Liu X. (2019). Chemical Characterization and Gastroprotective Effect of an Isolated Polysaccharide Fraction from *Bletilla Striata* against Ethanol-Induced Acute Gastric Ulcer. Food Chem. Toxicol. 131, 110539. 10.1016/j.fct.2019.0504710.1016/j.fct.2019.05.047 31158404

[B26] Zhu H. Dai O. Zhou F. Yang L. Liu F. Liu Y. (2021). Discovery of Bletillain, an Unusual Benzyl Polymer with Significant Autophagy-Inducing Effects in A549 Lung Cancer Cells through the Akt/GSK-3β/β-Catenin Signaling Pathway. Bioorg. Chem. 117, 105449. 10.1016/j.bioorg.2021.105449 34736136

