# Supplementary material for: Bioactive Bibenzyl Enantiomers From the Tubers of Bletilla striata
Source: Front Chem. 2022 Jun 9;10:911201. doi: 10.3389/fchem.2022.911201 (PMC9218944; doi:10.3389/fchem.2022.911201)
Supplement: Supplementary file 1 [file DataSheet1.docx]

**Bioactive bibenzyl enantiomers from the tubers of *Bletilla striata***

*Mei Zhou^1,2✝^, Sai Jiang^1,3✝^, Changfen Chen^1^, Jinyu Li^1,2^, Huayong Lou^1,2^, Mengyun Wang^3^, Gezhou Liu^1^, Hanfei Liu^1,2^, Ting* *Liu^4^,* and Weidong Pan^1,2^,**

*^1^School of Basic Medical Sciences/State Key Laboratory of Functions and Applications of Medicinal Plants, Guizhou Medical University, Guiyang 550025, PR China, ^2^The Key Laboratory of Chemistry for Natural Products of Guizhou Province and Chinese Academy of Sciences, Guiyang 550014, PR China, ^3^TCM and Ethnomedicine Innovation & Development International Laboratory, Innovative Materia Medica Research Institute, School of Pharmacy, Hunan University of Chinese Medicine, Changsha 410208, PR China,* *^4^Guizhou Provincial Key Laboratory of Pharmaceutics, Guizhou Medical University, Guiyang 550001, PR China*

^*^Correspondence:

*Ting Liu*

[liuting@gmc.edu.cn](mailto:liuting@gmc.edu.cn) (T. Liu)

*Weidong Pan*

wdpan@163.com (W.-D. Pan)

*^✝^*These authors contribute equally to this paper

Content

[FIGURE │ S1](#_Toc32000) ^[1](#_Toc32000)^[H NMR spectrum of compound 1 in DMSO-](#_Toc32000)*[d](#_Toc32000)*[6](#_Toc32000) [(600 MHz). 3](#_Toc32000)

[FIGURE │ S2](#_Toc7234) ^[13](#_Toc7234)^[C NMR spectrum of compound 1 in DMSO-](#_Toc7234)*[d](#_Toc7234)*[6](#_Toc7234) [(150 MHz). 3](#_Toc7234)

[FIGURE │ S3 HSQC spectrum of compound 1. 4](#_Toc20877)

[FIGURE │ S4 HMBC spectrum of compound 1. 4](#_Toc7784)

[FIGURE │ S5 HRESIMS spectrum of compound 1. 5](#_Toc16334)

[FIGURE │ S6 The experimental ECD curves of compound 1. 5](#_Toc6441)

[FIGURE │ S7 The UV spectrum of compound 1. 5](#_Toc5156)

[FIGURE │ S8 The FT-IR spectrum of compound 1. 6](#_Toc3841)

[FIGURE │ S9](#_Toc6192) ^[1](#_Toc6192)^[H NMR spectrum of compound 2 in methanol-](#_Toc6192)*[d](#_Toc6192)*[4](#_Toc6192) [(400 MHz). 6](#_Toc6192)

[FIGURE │ S10](#_Toc6079) ^[13](#_Toc6079)^[C NMR spectrum of compound 2 in methanol-](#_Toc6079)*[d](#_Toc6079)*[4](#_Toc6079) [(100 MHz). 7](#_Toc6079)

[FIGURE │ S11 HSQC spectrum of compound 2. 7](#_Toc28276)

[FIGURE │ S12 HMBC spectrum of compound 2. 8](#_Toc13854)

[FIGURE │ S13 HRESIMS spectrum of compound 2. 8](#_Toc22515)

[FIGURE │ S14 The experimental ECD curves of compound 2. 9](#_Toc13161)

[FIGURE │ S15 The UV spectrum of compound 2. 9](#_Toc7131)

[FIGURE │ S16 The FT-IR spectrum of compound 2. 9](#_Toc30966)

[FIGURE │ S17](#_Toc18817) ^[1](#_Toc18817)^[H NMR spectrum of compound 3 in methanol-](#_Toc18817)*[d](#_Toc18817)*[4](#_Toc18817) [(400 MHz). 10](#_Toc18817)

[FIGURE │ S18](#_Toc24249) ^[13](#_Toc24249)^[C NMR spectrum of compound 3 in methanol-](#_Toc24249)*[d](#_Toc24249)*[4](#_Toc24249) [(100 MHz). 10](#_Toc24249)

[FIGURE │ S19 SHSQC spectrum of compound 3. 11](#_Toc3893)

[FIGURE │ S20 HMBC spectrum of compound 3. 11](#_Toc24390)

[FIGURE │ S21 HRESIMS spectrum of compound 3. 12](#_Toc29878)

[FIGURE │ S22 The experimental ECD curves of compound 3. 12](#_Toc25840)

[FIGURE │ S23 The UV spectrum of compound 3. 12](#_Toc32337)

[FIGURE │ S24 The FT-IR spectrum of compound 3. 13](#_Toc16627)

[TABLE │ S1 Minimum inhibitory concentrations (MICs) for compounds 1-3. 13](#_Toc9712)


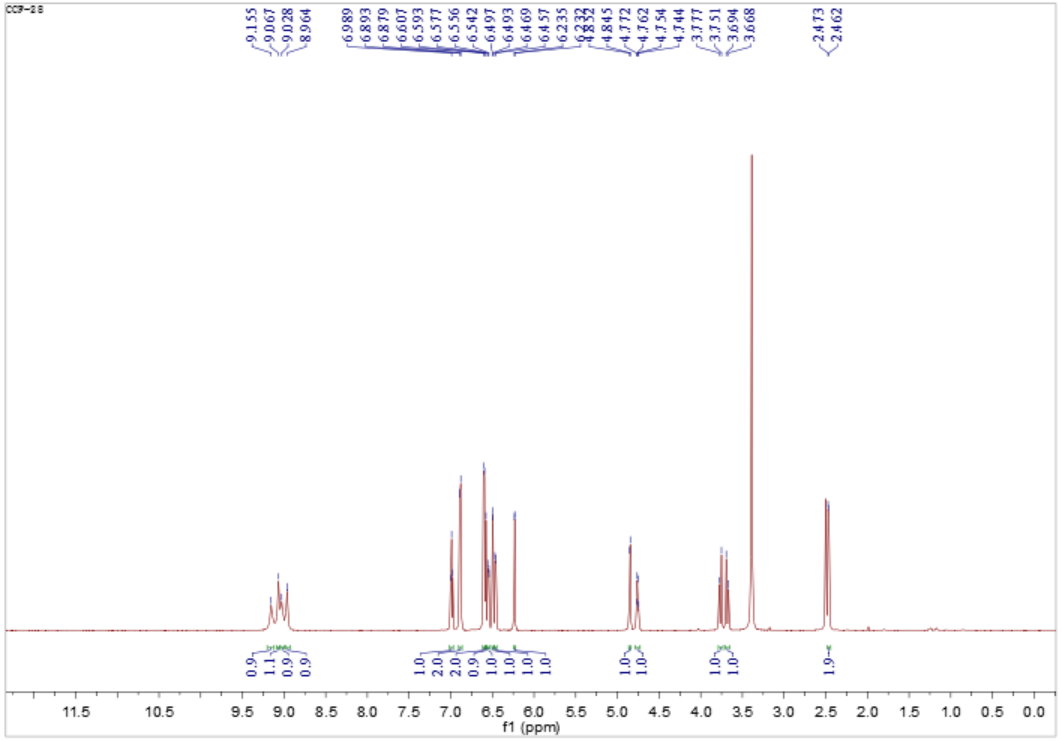


## FIGURE **│** **S1** ^1^H NMR spectrum of compound **1** in DMSO-*d*_6_ (600 MHz).


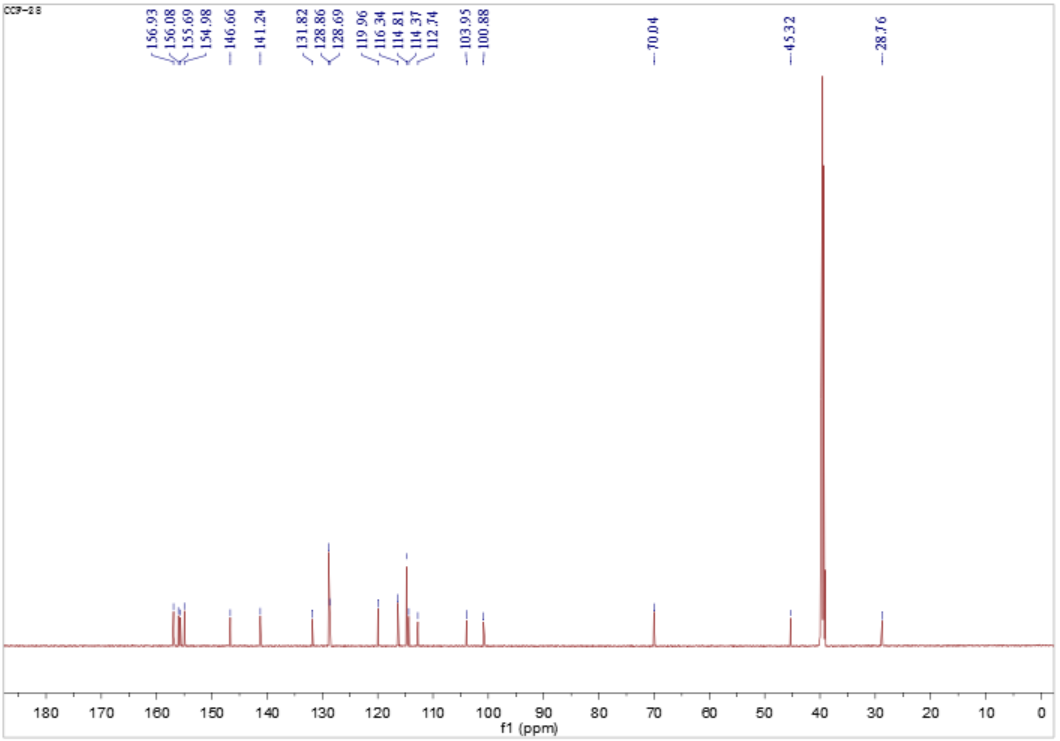


## FIGURE **│** **S2** ^13^C NMR spectrum of compound **1** in DMSO-*d*_6_ (150 MHz).


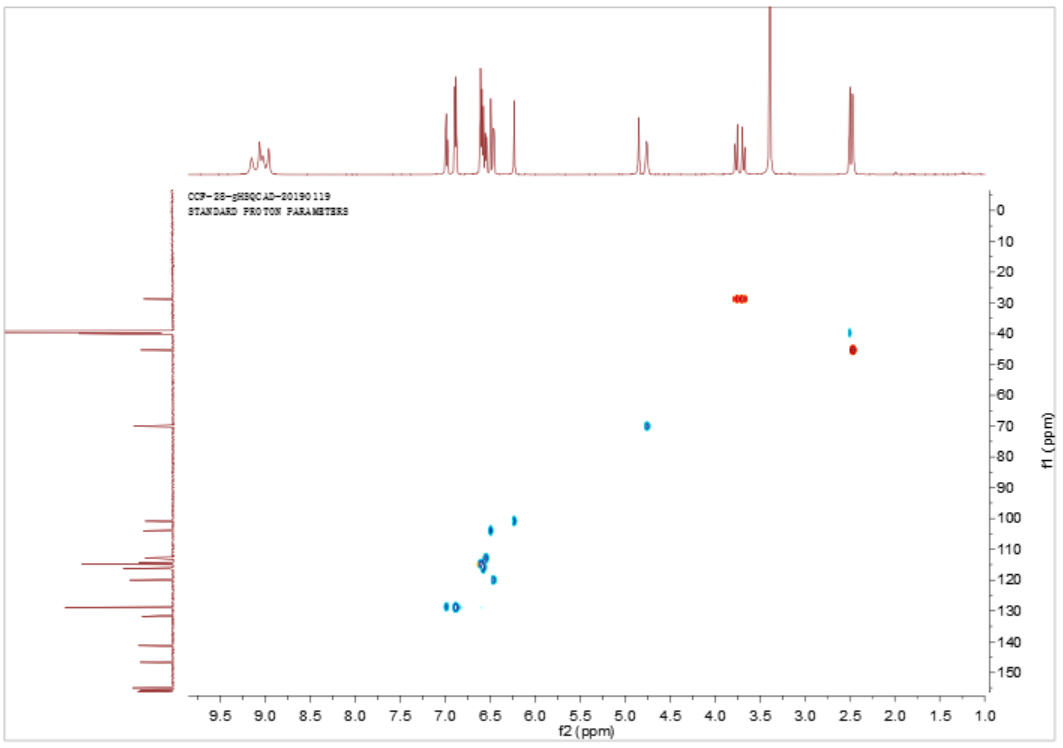


## FIGURE │ S3 HSQC spectrum of compound 1.


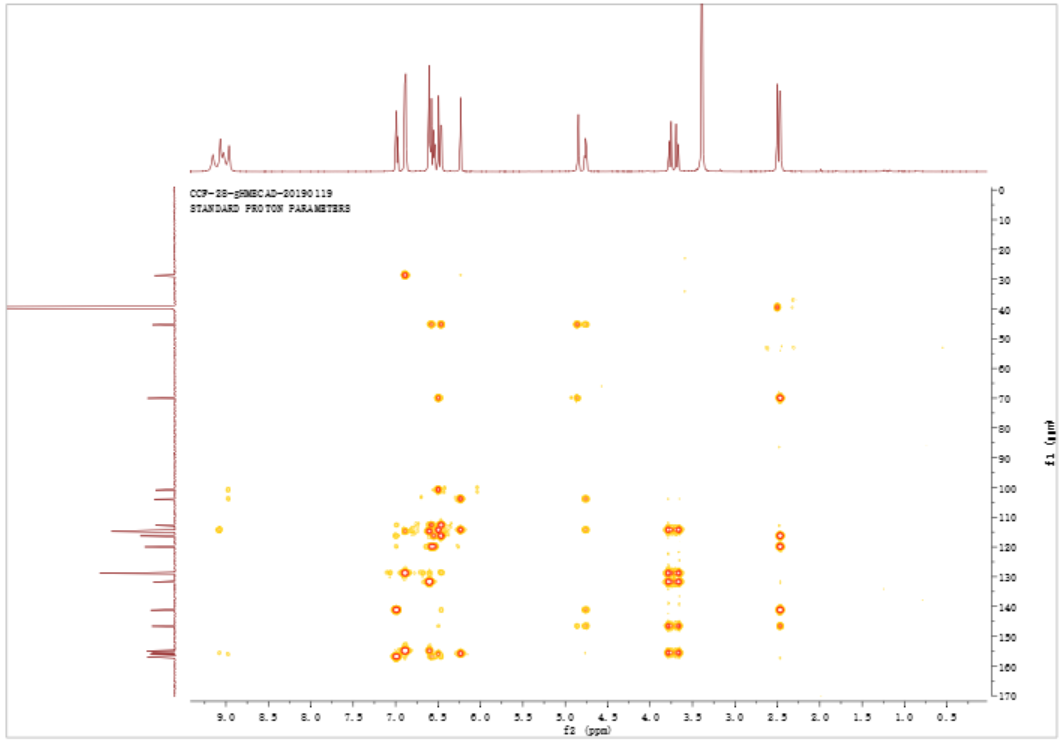


## FIGURE │ S4 HMBC spectrum of compound 1.

## FIGURE │ S5 HRESIMS spectrum of compound **1**.


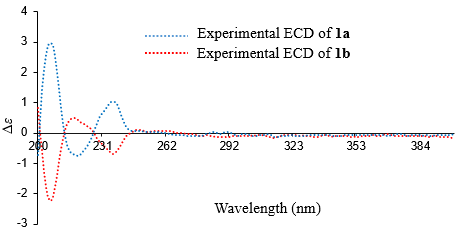


## FIGURE │ S6 The experimental ECD curves of compound **1**.


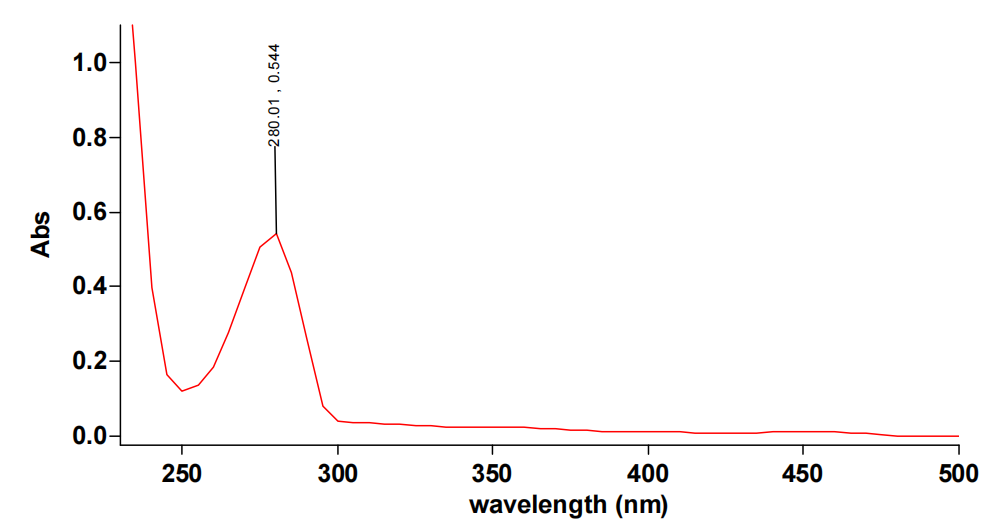


## FIGURE **│** S7 The UV spectrum of compound 1.


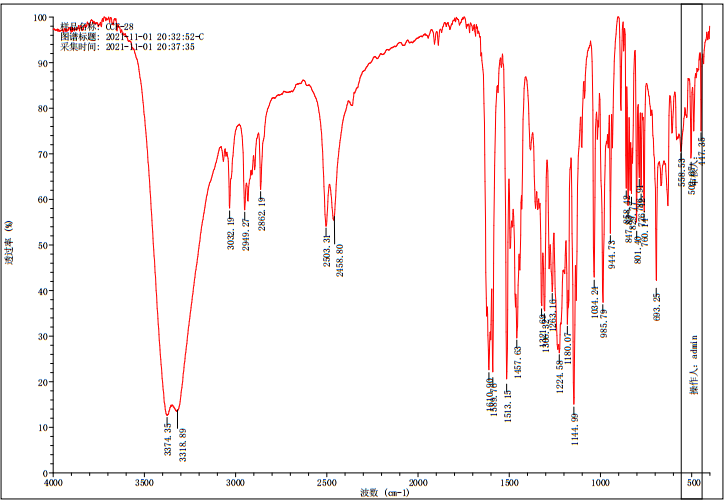


## FIGURE **│** S8 The FT-IR spectrum of compound 1.


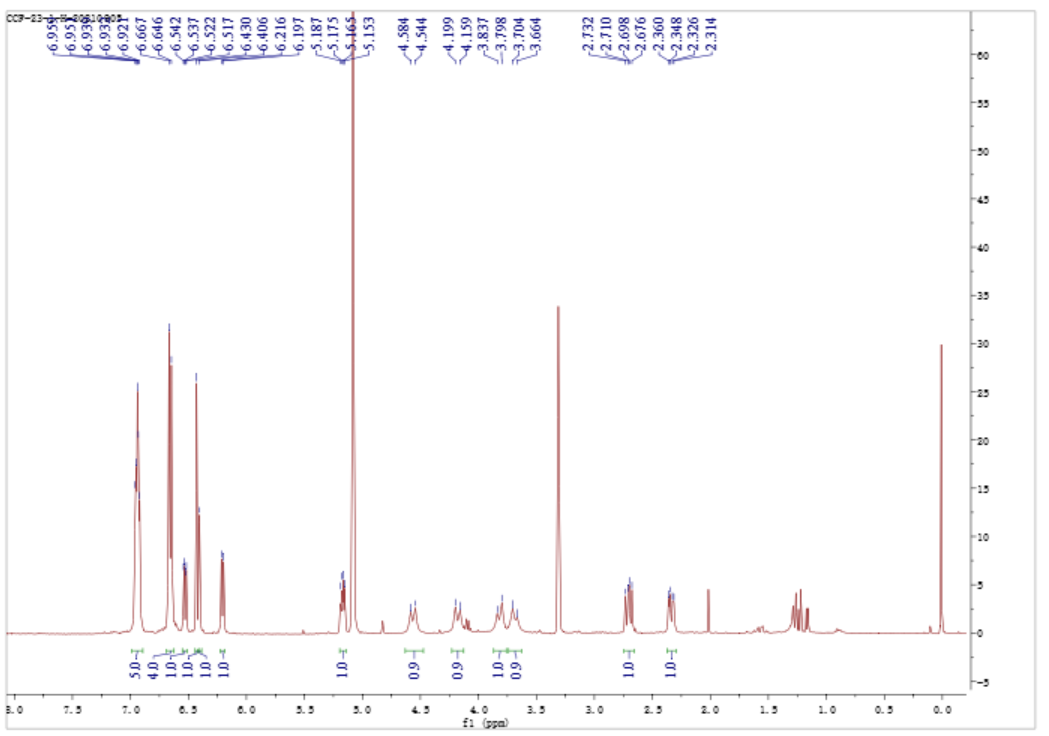


## FIGURE **│** S9 ^1^H NMR spectrum of compound 2 in methanol-*d*_4_ (400 MHz).


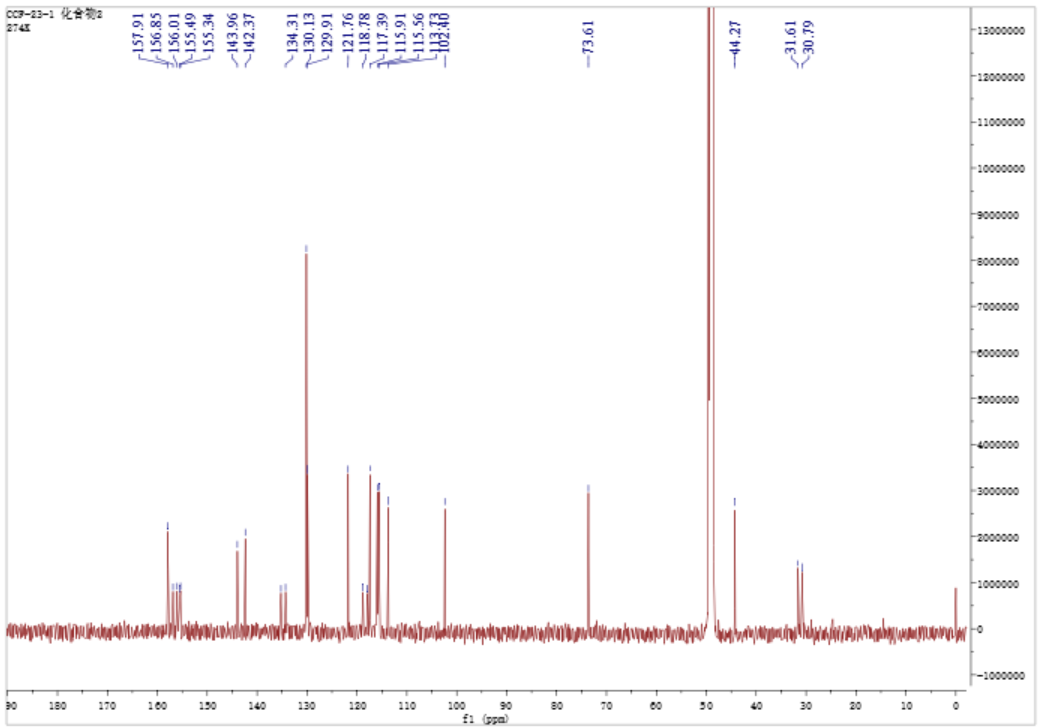


## FIGURE **│** S10 ^13^C NMR spectrum of compound **2** in methanol-*d*_4_ (100 MHz).


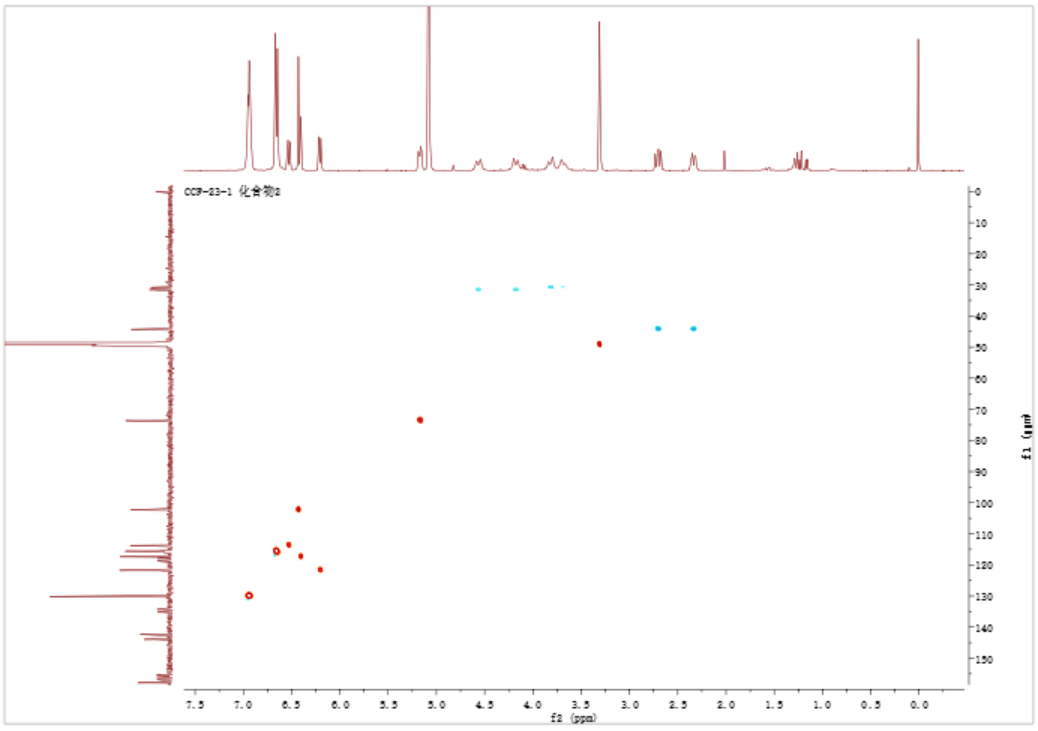


## FIGURE **│** S11 HSQC spectrum of compound 2.


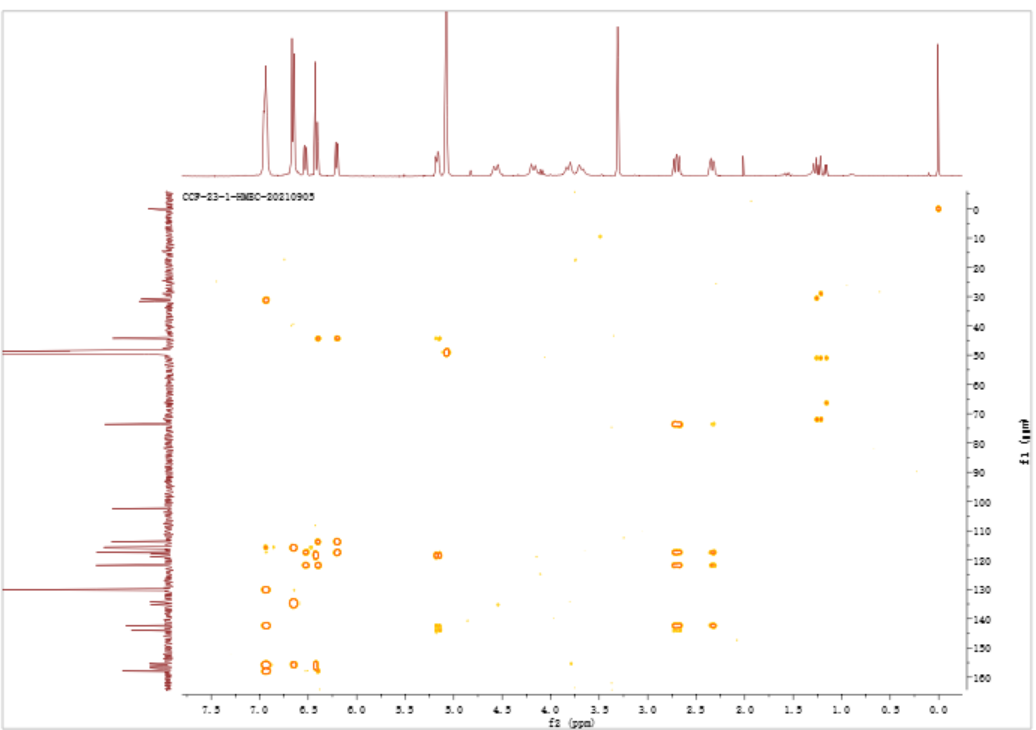


## FIGURE **│** S12 HMBC spectrum of compound 2.

## FIGURE **│** S13 HRESIMS spectrum of compound 2.


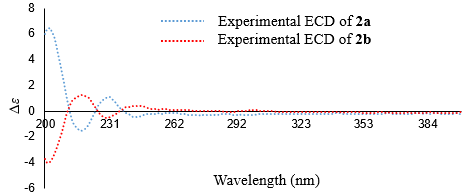


## FIGURE **│** S14 The experimental ECD curves of compound 2.


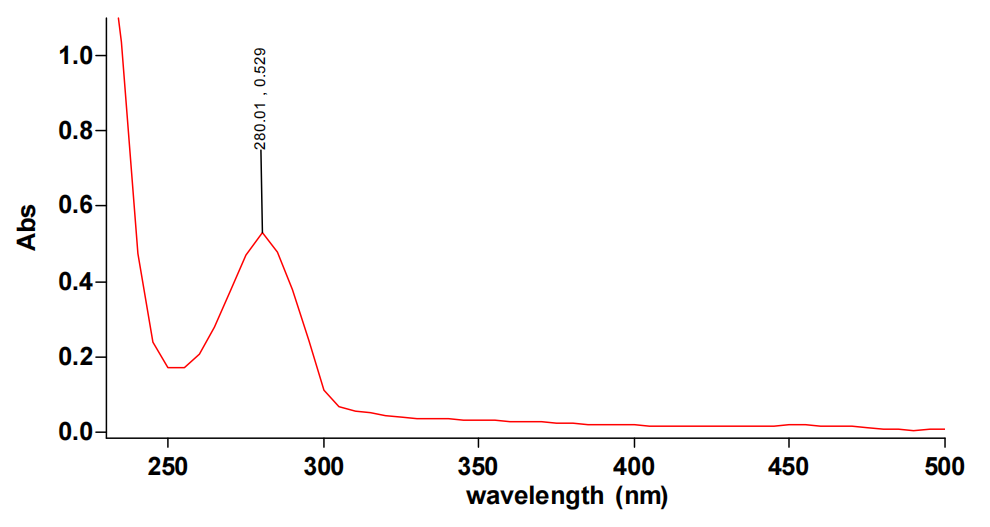


## FIGURE **│** S15 The UV spectrum of compound 2.


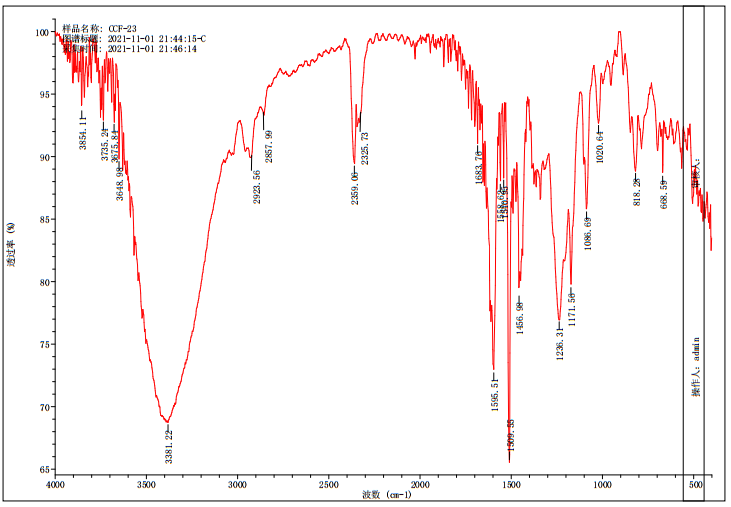


## FIGURE **│** S16 The FT-IR spectrum of compound 2.


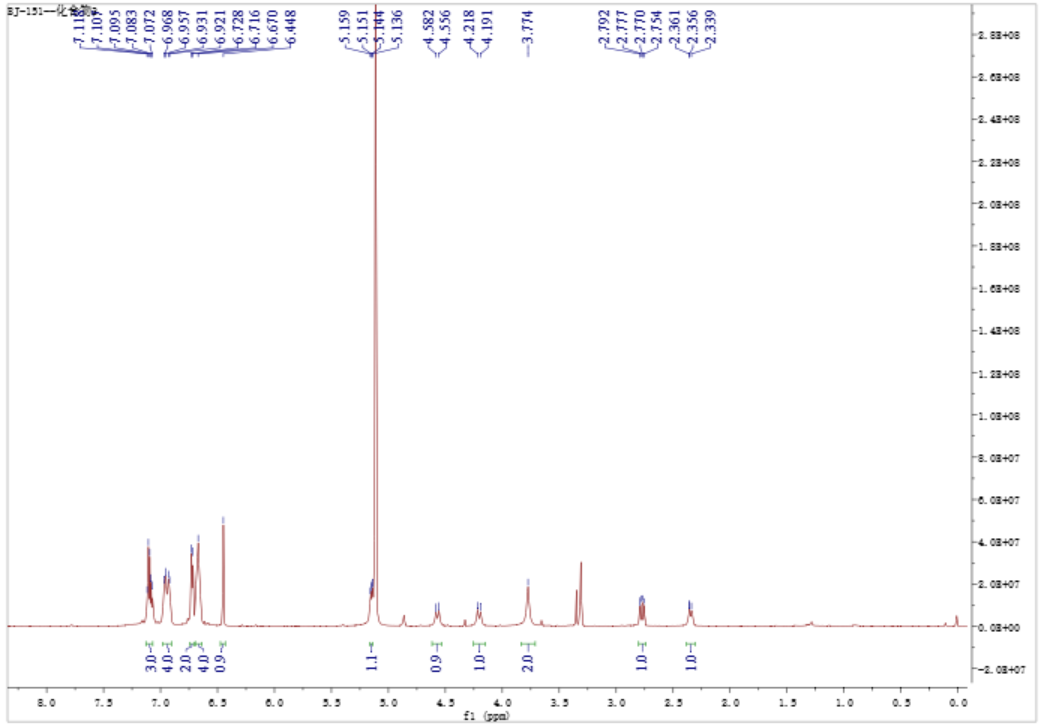


## FIGURE **│** S17 ^1^H NMR spectrum of compound 3 in methanol-*d*_4_ (400 MHz).


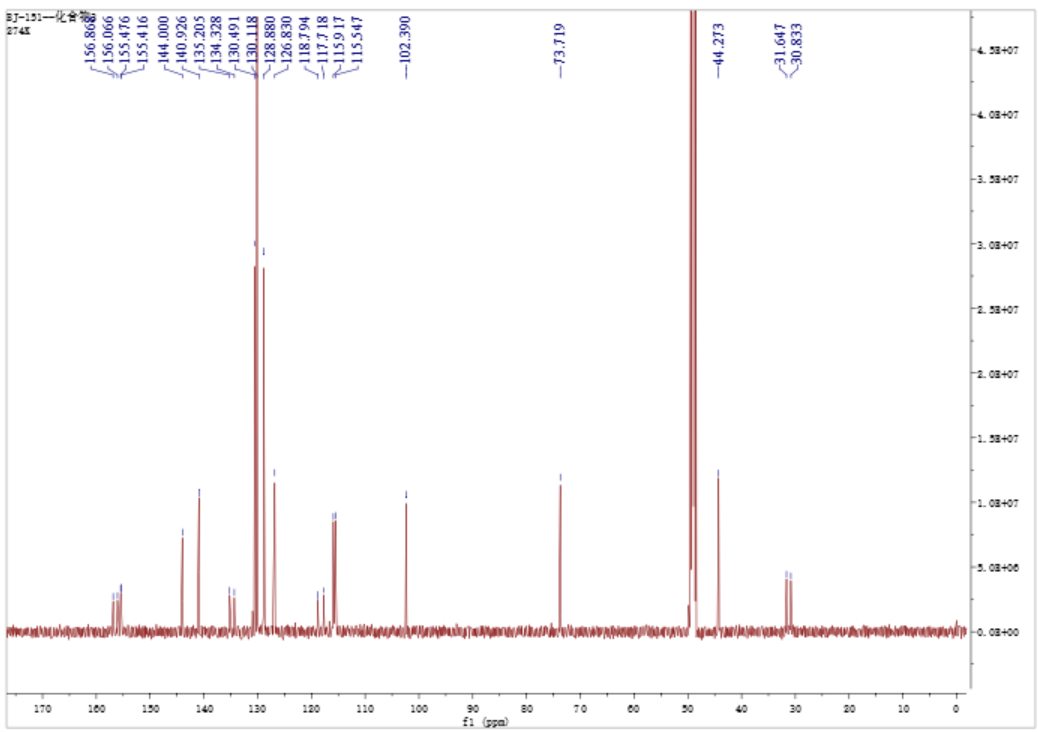


## FIGURE **│** S18 ^13^C NMR spectrum of compound 3 in methanol-*d*_4_ (100 MHz).


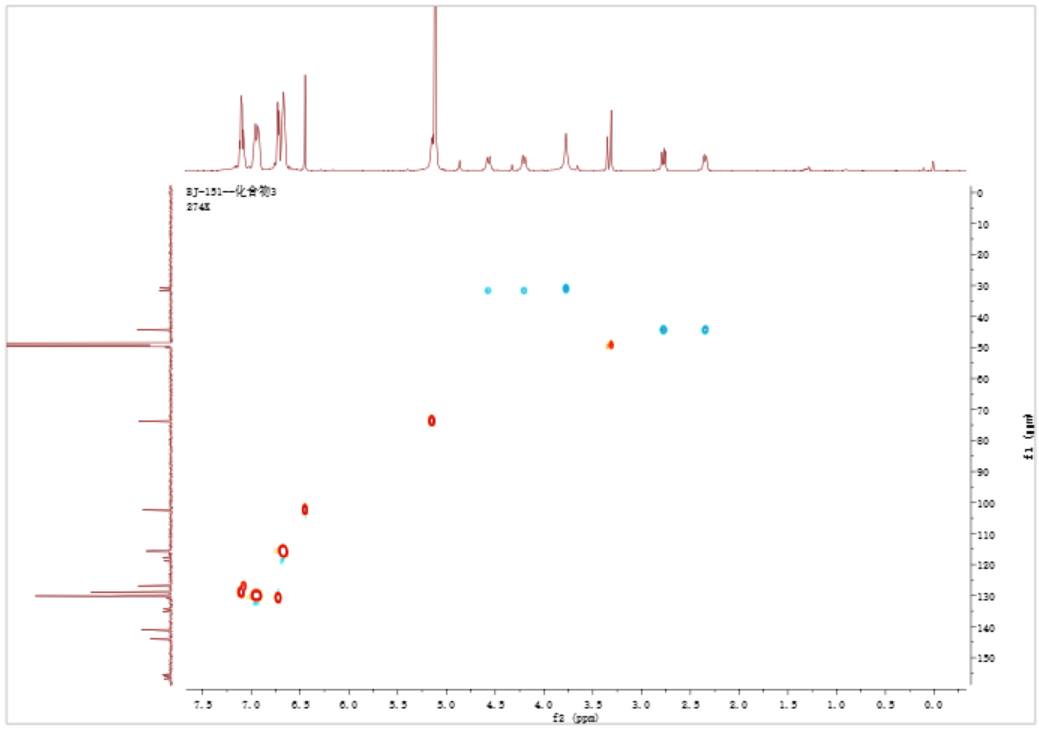


## FIGURE **│** S19 SHSQC spectrum of compound 3.


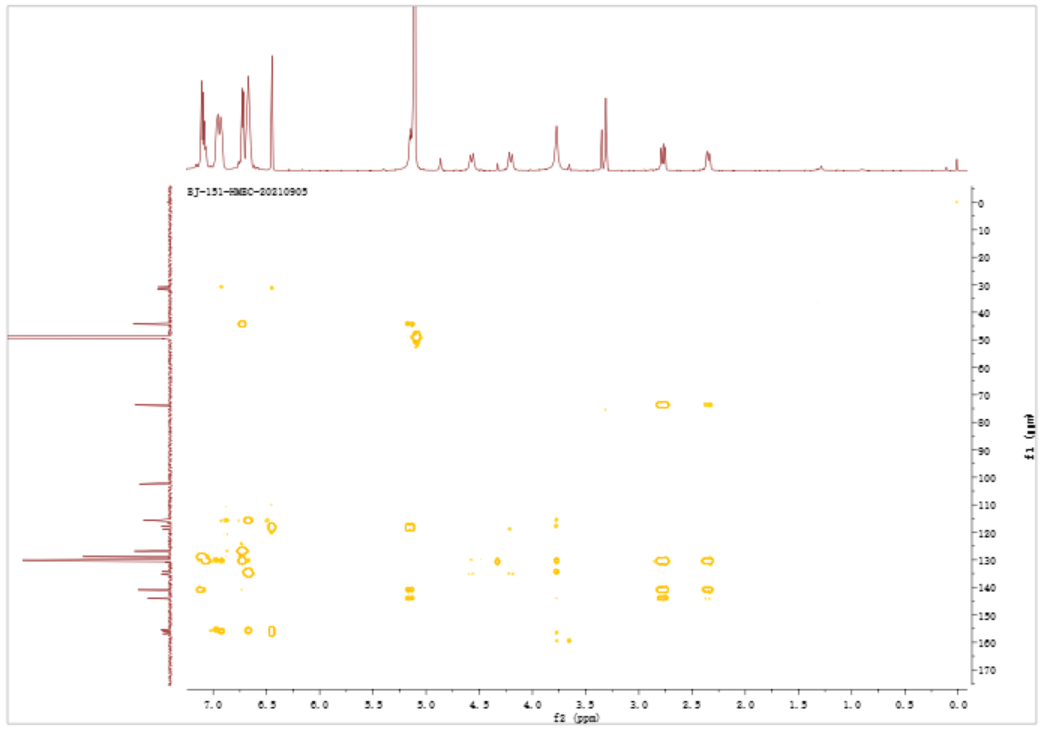


## FIGURE **│** S20 HMBC spectrum of compound 3.

## FIGURE **│** S21 HRESIMS spectrum of compound 3.


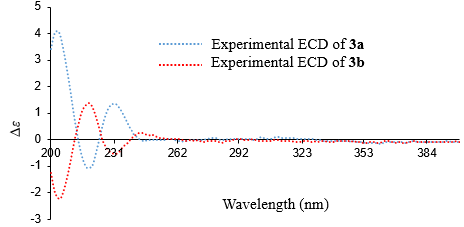


## FIGURE **│** S22 The experimental ECD curves of compound 3.


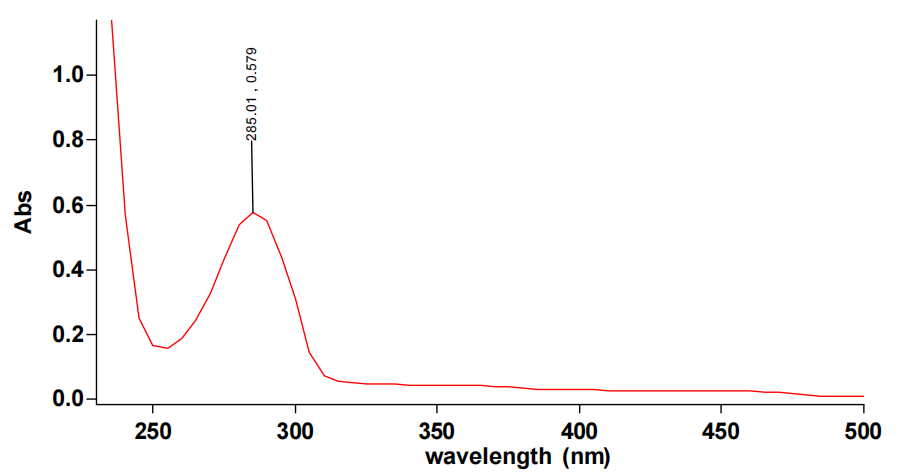


## FIGURE **│** S23 The UV spectrum of compound 3.


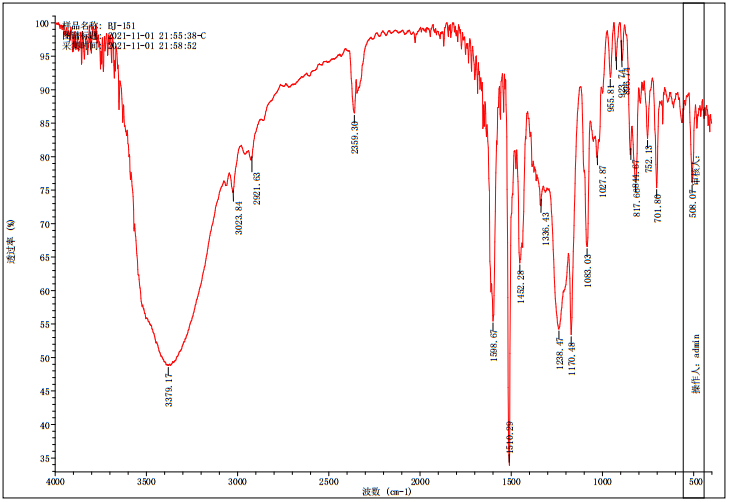


## FIGURE **│** S24 The FT-IR spectrum of compound 3.

## TABLE **│ S1** Minimum inhibitory concentrations (MICs) for compounds **1-3**.

|  | MIC (μg/mL) | | | |
| --- | --- | --- | --- | --- |
| Compounds | *S*. *aureus* ATCC 6538 | *Bacillus subtilis* ATCC 6051 | *Methicillin-resistant S*. *aureus* ATCC 43300 | *Escherichia coli* ATCC 11775 |
| **1a** | >420 | >420 | >420 | >420 |
| **1b** | >420 | >420 | >420 | >420 |
| **2a** | >420 | >420 | >420 | >420 |
| **2b** | >420 | >420 | >420 | >420 |
| **3a** | 52 | 105 | 105 | >420 |
| **3b** | 52 | 105 | 105 | >420 |
| oxacillin | 0.0078 | 1.25 | 3.13 | 137.5 |
