# Supplementary material for: Bioactive Bibenzyl Enantiomers From the Tubers of Bletilla striata
Source: Front Chem. 2022 Jun 9;10:911201. doi: 10.3389/fchem.2022.911201 (PMC9218944; doi:10.3389/fchem.2022.911201)
Supplement: Supplementary file 2 [file Image1.pdf]

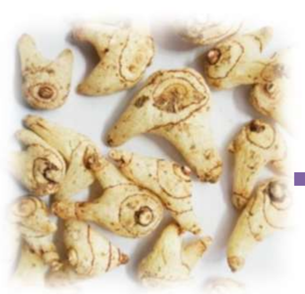

Bletilla striata **(Thunb.) Reichb.f.**

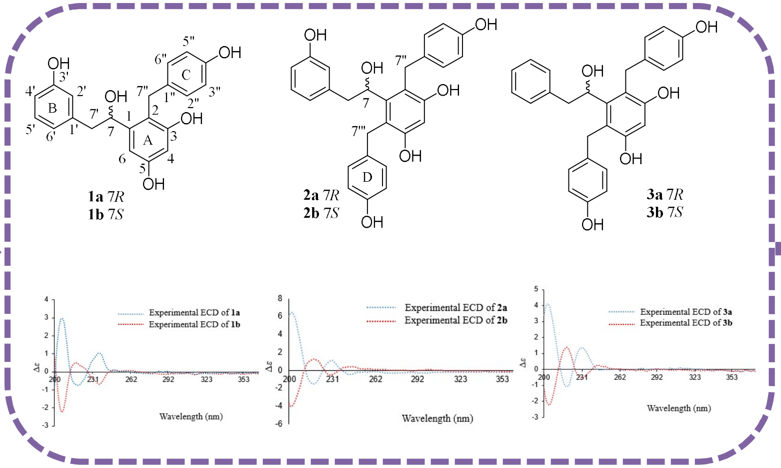

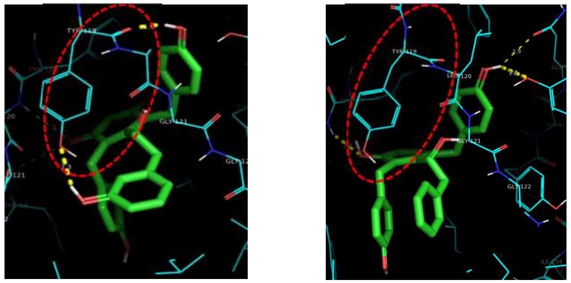

Three-dimensional molecular docking model of TNF-*α* for compounds 2 and 3

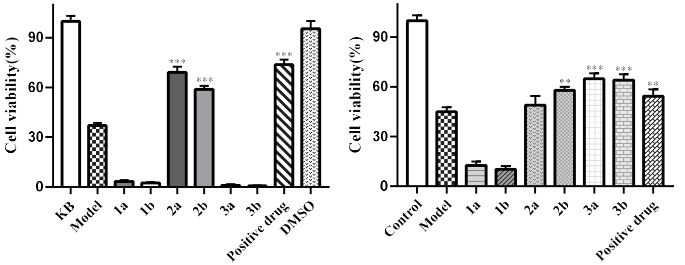

Anti-TNF-*α* activities of compounds 1-3. **Neuroprotective activities of compounds 1-3**
